# Targeting the coronavirus SARS-CoV-2: computational insights into the mechanism of action of the protease inhibitors lopinavir, ritonavir and nelfinavir

**DOI:** 10.1038/s41598-020-77700-z

**Published:** 2020-12-01

**Authors:** Giovanni Bolcato, Maicol Bissaro, Matteo Pavan, Mattia Sturlese, Stefano Moro

**Affiliations:** Molecular Modeling Section (MMS), Department of Pharmaceutical and Pharmacological Sciences, University of Padova, Via Marzolo 5, 35131 Padova, Italy

**Keywords:** Drug discovery, Medicinal chemistry, Infectious diseases

## Abstract

Coronavirus SARS-CoV-2 is a recently discovered single-stranded RNA betacoronavirus, responsible for a severe respiratory disease known as coronavirus disease 2019, which is rapidly spreading. Chinese health authorities, as a response to the lack of an effective therapeutic strategy, started to investigate the use of lopinavir and ritonavir, previously optimized for the treatment and prevention of HIV/AIDS viral infection. Despite the clinical use of these two drugs, no information regarding their possible mechanism of action at the molecular level is still known for SARS-CoV-2. Very recently, the crystallographic structure of the SARS-CoV-2 main protease (M^pro^), also known as C30 Endopeptidase, was published. Starting from this essential structural information, in the present work we have exploited supervised molecular dynamics, an emerging computational technique that allows investigating at an atomic level the recognition process of a ligand from its unbound to the final bound state. In this research, we provided molecular insight on the whole recognition pathway of Lopinavir, Ritonavir, and Nelfinavir, three potential C30 Endopeptidase inhibitors, with the last one taken into consideration due to the promising in-vitro activity shown against the structurally related SARS-CoV protease.

## Introduction

Coronavirus SARS-CoV-2, previously known as 2019-nCoV, is a recently discovered single-stranded RNA (ssRNA) betacoronavirus, responsible for a severe pathological condition known as coronavirus disease 2019 (COVID-19)^1^. Since it was first identified in December 2019, this novel coronavirus has rapidly spread all around the world, being since now responsible for the death of more than one million of people, which have lost their lives due to a severe respiratory illness^2^.

The first outbreak of this new disease originally took place in the city of Wuhan (China), rapidly spreading in the southeast of Asia and, recently, in other continents like Europe, North America and Africa^1^. The astonishing rate at which COVID is expanding compared to previous coronavirus related diseases (SARS-CoV and MERS-CoV), in conjunction with the absence of approved drugs or effective therapeutic approaches for its treatment, has gathered the attention of the international community, which is promoting a cooperative effort to face this emergency^3,4^. On January 2020 indeed, the International Health Regulations Emergency Committee of the World Health Organization declared the outbreak a “public health emergency of international concern” in responding to SARS-CoV-2.

Unfortunately, the timeline characterizing a typical drug discovery process badly couples with the urgency of finding a cure for the already infected patients as rapidly as possible. In this kind of scenario, it is of paramount importance to accelerate the early stages of the drug discovery process for COVID-19 treatment, and for all possible future emergencies^5^.

The early isolation of the SARS-CoV-2 genome from ill patients represented a first crucial outcome, making it possible to highlight an important sequence identity (~ 80% of conserved nucleotides) with respect to the original SARS-CoV epidemic virus^6^. In light of this similarity, some therapeutic strategies could be inherited from other genetically related CoV diseases.

A possible target is for example represented by structural viral proteins, therefore interfering with the assembly and the internalization of the pathogen into the host, which was shown to occur also in this case through the Angiotensin-converting enzyme II (ACE2) receptor. From this perspective, the development of a vaccine is desirable, and it is foreseen that the first candidates will be advanced to clinical phase I around mid-2020^7–9^.

In the meantime, however, a great effort involves the targeting of non-structural viral proteins which are instead essential for the viral replication and the maturation processes, thus representing a crucial and specific target for anti-COVID drug development^3,10^. In this regard, the crystallographic structure of the SARS-CoV-2 main protease (M^pro^), also known as C30 Endopeptidase, was elucidated and made available to the scientific community with impressive timing, just a few weeks after the first COVID-19 outbreak (PDB ID: 6LU7). The structural characterization of the protease, which shares 96.1% of its sequence with those of SARS-CoV, has revealed a highly conserved architecture of the catalytic binding site.

As a result, structure-based drug discovery techniques (SBDD) can now be applied to efficiently speed up the rational identification of putative M^pro^ inhibitors or to drive the repurposing process of known therapy. This latter route is particularly attractive, as it allows to significantly shrink the time required to access the first phases of clinical trials, without compromising patient safety. A multitude of research groups has begun to apply computational approaches, such as molecular docking based virtual screening (VS), to evaluate already approved FDA approved drugs against the aforementioned viral protease^11–14^. Many of these studies have found convergence in suggesting compounds inhibitors of the human immunodeficiency viruses (HIV) as possible anti-COVID candidates; this is surprising considering the important structural differences exiting among these two homologous enzymes. The repositioning of HIV antiviral drugs for the treatment of coronavirus infections found, however, a foundation in the scientific literature of the past 20 years. Some of these compounds have therefore been experimentally investigated, showing promising activity, both in the case of SARS-CoV and MERS-CoV outbreak^15,16^.

Moreover, at least three randomized clinical trials are currently been held in China in order to evaluate the therapeutic efficacy of Lopinavir and Ritonavir, a combination of HIV protease inhibitors, in COVID-19 treatment^7^. In this perspective and preliminary computational research, we took advantage of the recently solved crystallographic structure of SARS-CoV-2 M^pro^ to perform a cutting edge in-silico investigation.

**Figure 1:**
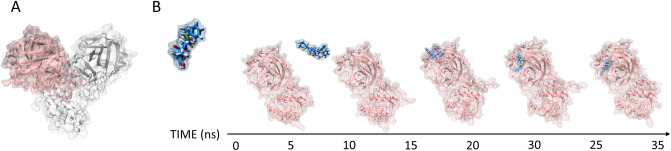
The crystallographic structure of SARS-CoV-2 C30 Endopeptidase exploited in our computational investigation (PDB ID: 6LU7) is reported in Panel **A**. The two different monomers composing the homodimeric proteases are depicted using different colors (i.e. pink and white respectively for monomer **A** and **B**). As represented on Panel **B**, only one chain (monomer **A**) was exploited in our SuMD protocol to describe the putative inhibitor binding mechanism.

Supervised molecular dynamics (SuMD), an emerging technique allowing to investigate at an atomic level of detail the molecular recognition process, was exploited to characterize the putative binding mechanism of three HIV protease inhibitors^17–19^. In detail, along with the aforementioned combination of Lopinavir and Ritonavir, also Nelfinavir was taken into consideration, due to the promising in-vitro activity shown by this compound against the structurally related SARS-CoV protease^20^. SuMD protocol implements a tabu-like algorithm that controls the sampling of short unbiased MD trajectories, each of which hundreds of picoseconds (ps) long. In detail, simulation steps are accepted only when describing a ligand approaching a known binding site, otherwise, the simulation is discharged and restarted from the previous coordinate set. The combination of all productive SuMD simulation steps represents, therefore, a putative molecular recognition trajectory collected, differently from brute force MD, in a very competitive computational time not exceeding the nanoseconds (ns) timescale (Fig. 1). Contrary to molecular docking, SuMD simulations fully consider both the flexibility characterizing the protein target during the binding event and the contribution played by water molecules during the recognition. Moreover, the study is not limited to a possible final state but allows peeking dynamically at the whole process of recognition, also identifying putative metastable binding sites.

## Results

The combination of the structurally related antiviral protease inhibitor Lopinavir and Ritonavir, commercially known with the name Kaletra, represent an effective therapeutic weapon ensuring an adequate and durable suppression of viral load in HIV positive patients. The synergistic coadministration of these two compounds exploits low-dosage concentration of Ritonavir which, inhibiting the metabolic inactivation of Lopinavir, acts as a pharmacokinetic enhancer^21^. Following a preliminary favorable clinical response in SARS-CoV related diseases, the combination of the drug is currently under investigation also against SARS-CoV-2, with at least three randomized clinical trials undergoing with Chinese infected patients^15^. In our computational study, we considered Lopinavir and Ritonavir as two independent inhibitors, performing separate SuMD binding simulations, which results are herein reported an analyzed.

**Figure 2:**
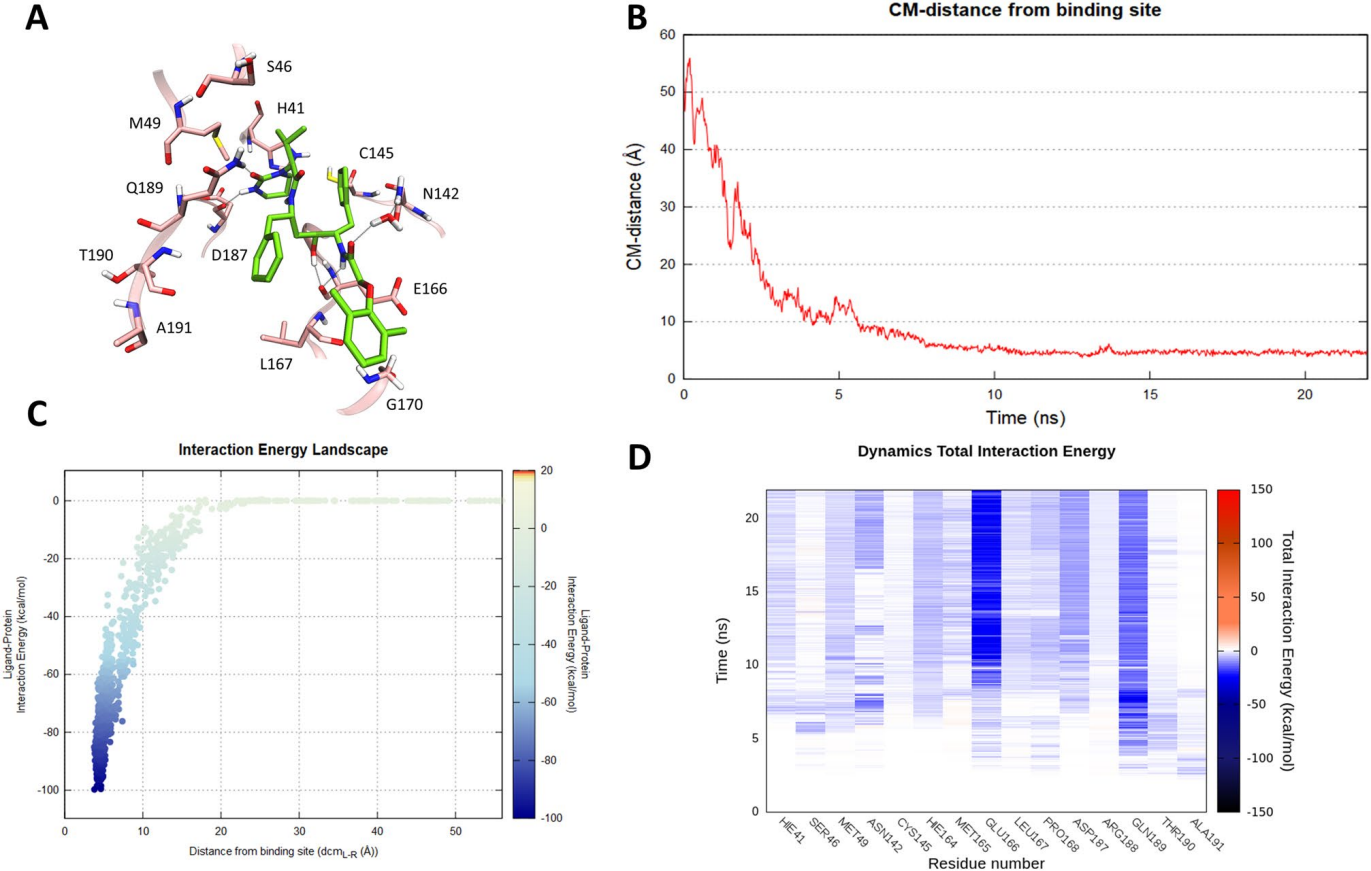
This panel summarizes the recognition pathway of Lopinavir against the SARS-CoV-2 main protease. (**A**) Lopinavir conformation sampled in the last frame of the SuMD trajectory (green-colored molecule). The residues surrounding the binding site are reported in pink color. (**B**) Distance between the ligand center of mass (Cm) and the catalytic binding site of the M^pro^ during the SuMD simulation. (**C**) Interaction Energy Landscape describing the protein–ligand recognition process; values are arranged according to the distances between ligand and protein target mass centers. (**D**) Dynamic total interaction energy (electrostatic + vdW) computed for most contacted M^pro^ residues.

As highlighted in Fig. 2 (Panel B) about 20 ns proved to be sufficient to sample a putative Lopinavir recognition trajectory with SARS-CoV-2 protease. At a distance of about 15 Å from the binding site, the first molecular contacts are recorded (Fig. 2—Panel C, D and Video 1), which guide the subsequent accommodation of the ligand into the catalytic site. The predicted final state is stabilized by a double hydrogen bond interaction with residue Glu166 backbone, tightly anchoring the inhibitor (Fig. 2—Panel A). This strong and persistent interaction (Fig. 2—Panel B) is known to be crucial in many SARS-CoV complexes and moreover, was also found to stabilize the covalent peptidomimetic compound crystallized in the recently published SARS-CoV-2 M^pro^ structure. In addition, the cyclic urea moiety of Lopinavir mediates a hydrogen bond interaction with the side chain of Gln189, another residue whose importance has been elucidated by means of several SARS-CoV three-dimensional complexes.

**Figure 3:**
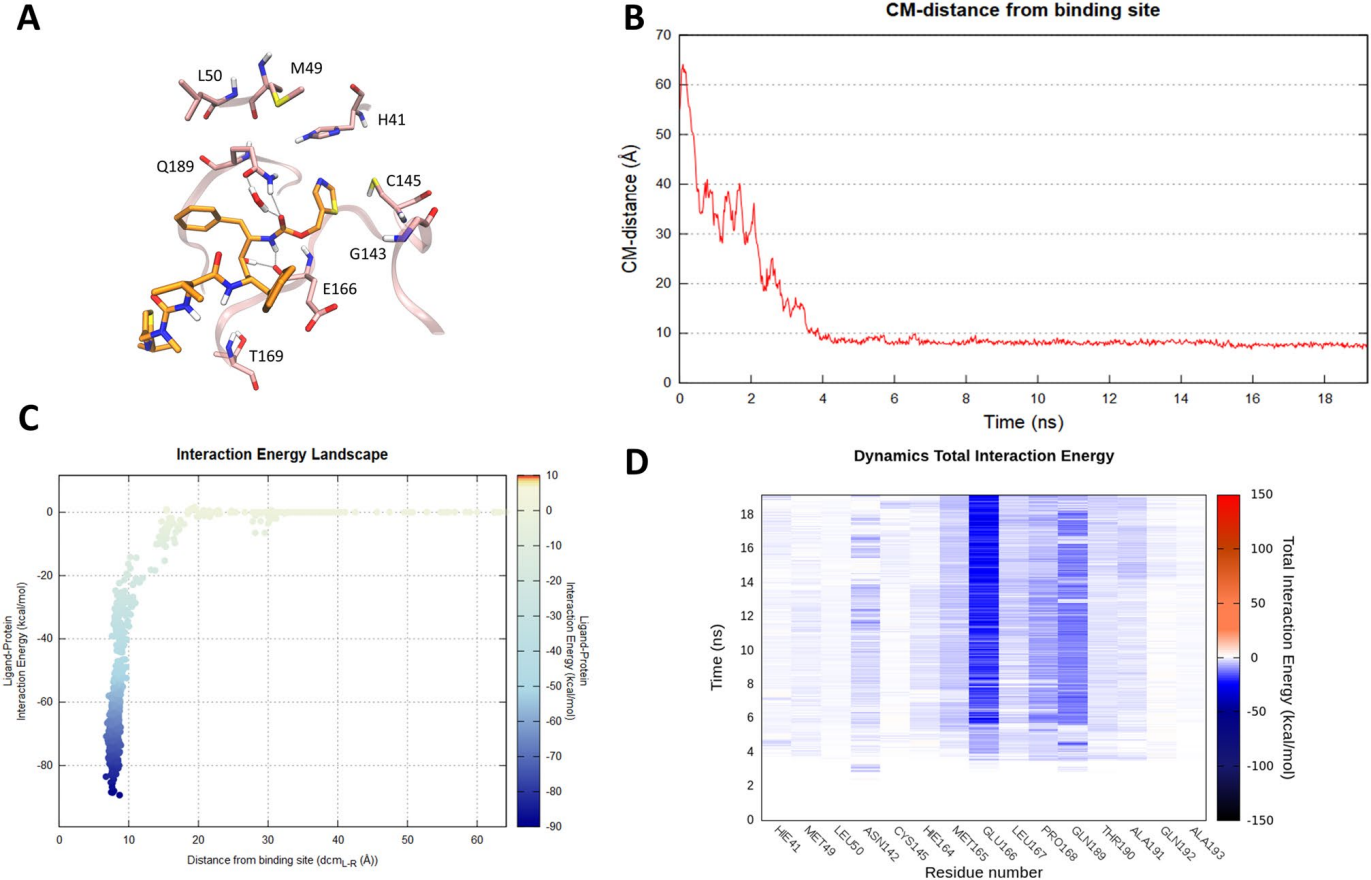
This panel summarizes the recognition pathway of Ritonavir against the SARS-CoV-2 main protease. (**A**) Ritonavir conformation sampled in the last frame of the SuMD trajectory (orange-colored molecule). The residues surrounding the binding site are reported in pink color. (**B**) Distance between the ligand center of mass (Cm) and the catalytic binding site of the M^pro^ during the SuMD simulation. (**C**) Interaction Energy Landscape describing the protein–ligand recognition process; values are arranged according to the distances between ligand and protein target mass centers. (**D**) Dynamic total interaction energy (electrostatic + vdW) computed for most contacted M^pro^ residues.

Despite the modest pharmacodynamic contribution made by Ritonavir in the combined formulation under investigation by the Chinese scientific community, in which the drugs act as a pharmacokinetic enhancer rather than a protease inhibitor, we still tried to elucidate its putative molecular recognition pathway. Also, in this case, 20 ns of SuMD simulation time were sufficient to sample a binding trajectory (Fig. 3—Panel B). Although some key interactions—i.e. hydrogen bond network with residue Glu166 and Gln189—are appreciable also in this final state (Fig. 3—Panel A, D and Video 2), a comparative analysis of the Interaction Energy Landscape graphs (Panel C of Figs. 2 and 3) suggests lower energy stability of the SuMD predicted binding mode, when compared with that characterizing Lopinavir. A reason could be seeking on the non-optimal accommodation of Ritonavir urea moiety, which floats outside the binding site exposed to the bulk solvent during all the simulation (Video 2).

**Figure 4:**
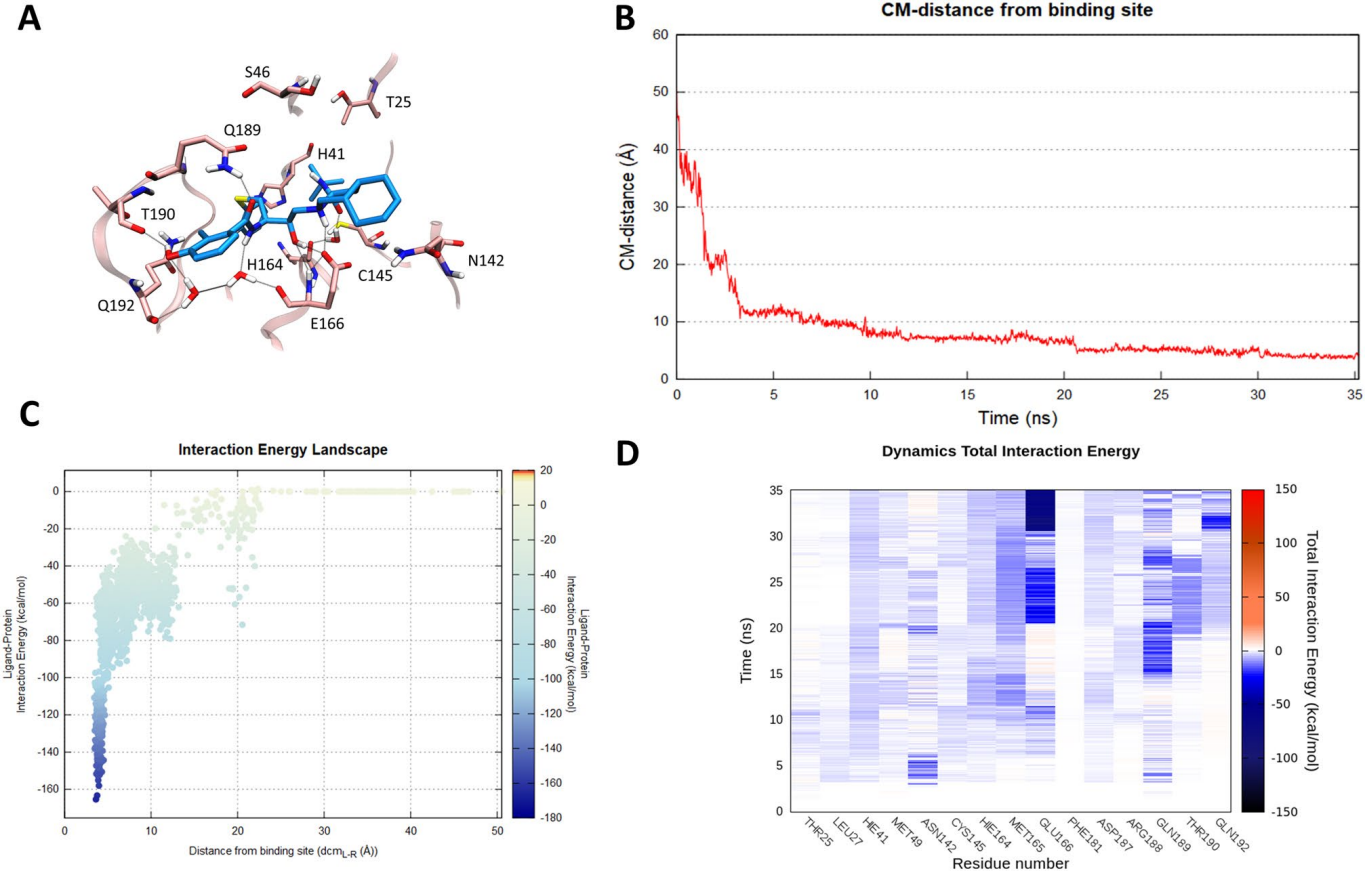
This panel summarizes the recognition pathway of Nelfinavir against the SARS-CoV-2 main protease. (**A**) Nelfinavir conformation sampled in the last frame of the SuMD trajectory (cyan-colored molecule). The residues surrounding the binding site are reported in pink color. (**B**) Distance between the ligand center of mass (Cm) and the catalytic binding site of the M^pro^ during the SuMD simulation. (**C**) Interaction Energy Landscape describing the protein–ligand recognition process; values are arranged according to the distances between ligand and protein target mass centers. (**D**) Dynamic total interaction energy (electrostatic + vdW) computed for most contacted M^pro^ residues.

In light of the promising experimental results shown by Nelfinavir, which milded the cytopathic effect induced by SARS-CoV infection strongly inhibiting the virus replication, we decided to computationally evaluate its possible molecular recognition mechanism also against SARS-CoV-2 protease. As reported in Fig. 4 (Panel B), a slightly longer SuMD simulation was necessary to fully describe a putative Nelfinavir binding trajectory. Once it has approached the vestibular region of the protease catalytic site, the ligand spends the first 20 ns negotiating the accommodation with a series of polar residues with which it mediates intermittent interactions, as highlighted in the interaction energy fingerprint (Fig. 4—Panel D, Video 3). The importance of this metastable site is also depicted in the Interaction Energy Landscape (IEL) graphic (Fig. 4—Panel C, Figure S3—Panel A and B), from which it is possible to notice a highly populated region presenting ligand–protein interaction energy comparable to the final states previously described for the other two inhibitors. The last 10 ns of the simulation were characterized by a series of conformational rearrangements, which resulted in an optimal Nelfinavir accommodation within the protease binding cleft stabilized through a dense hydrogen bond network, tightly anchoring the inhibitor to the protease. As shown in Fig. 4 (Panel A), SuMD predicted binding mode of Nelfinavir is characterized by great analogies with that of the originally crystallized covalent peptidomimetic compound. Residues His164, Glu166, Gln189, Thr190, and Gln196 mediate a series of directed or water-bridged hydrogen bonds interactions. Moreover, as highlighted in Fig. 4 (Panel D), on the last ns of the simulation a stabilizing salt bridge interaction occurs between the side chain of residue Glu166 and the octahydro-1H-isoquinoline charged moiety of Nelfinavir. Intriguingly, mutagenesis studies have corroborated the crucial role played by this residue. Mutation of Glu166 correlated therefore with the block of substrate-induced dimerization of the main protease, both in SARS-CoV and in MERS-CoV^22,23^.

## Discussion

In the last two decades, three major outbreaks of coronavirus-related diseasesSARS-CoV, MERS-CoV and ultimatelySARS-CoV-2 have been responsible for significant public health issues, along with dramatic social-economic consequences. The process of drug discovery often undergoes timelines which are difficult to reconcile with the urgency and the need to provide an effective therapeutic response to such an emergency health situation. Drug repurposing could represent a viable possibility, and this is the case for some anti-HIV compounds targeting SARS-CoV-2 C30 Endopeptidase. The molecular basis underneath their therapeutic action remains however often obscure. In this preliminary computational investigation, we have taken advantage of the recently published crystallographic structure of SARS-CoV-2 M^pro^ to investigate the putative binding mechanism of three antiviral compounds, previously designed as selective HIV protease inhibitors and now under investigation as anti-COVID-19 emergency treatments. SuMD protocol was in detail exploited to collect, for each of the three inhibitors, MD simulation describing the possible mechanism of molecular recognition, thus providing an atomistic insight to interpret their data of therapeutic efficacy. An interesting aspect is represented by the speed of this approach: a few days of calculation in a modest GPU cluster allowed to collect a multitude of simulations, from which it was possible to hypothesize the recognition mechanism of Lopinavir, Ritonavir, and Nelfinavir. An approach of this type, therefore, becomes crucial in all emergencies, making it possible to overcome the lack of structural data to guide and understand the possible repositioning of already approved drugs. In this particular case study, the SuMD protocol not only allowed to hypothesize a possible recognition method for each antiviral but also to advance some preliminary comparative considerations. Nelfinavir, in particular, showed the best fitting for the catalytic site of SARS-CoV-2 M^pro^, establishing an interactions network similar to those elucidated in the crystallographic complex for the covalent peptidomimetic compound N3. More specifically, the phenyl sulfanyl moiety of the protease inhibitor at the end of the simulation was completely buried within the hydrophobic sub-pocket S2, which is delimited by residues His41, Cys44, Met49 and Met165. The stabilizing vdW contribution mediated by these residues has been dynamically mapped during the entire simulation and it is appreciable in Figure S3. Encouragingly, a recent fragment crystallographic screening has highlighted how this site, precisely renamed “aromatic wheel”, consistently accommodates aromatic fragments mediating hydrophobic interactions with the surrounding residues^24^. Furthermore, Nelfinavir hydroxyl group engages a hydrogen bond interaction with the carbonyl backbone of Glu166, a key residue found to stabilize most of the aforementioned non-covalent fragments as well as many covalent peptidomimetic inhibitors. The optimal interactive network differentiating Nelfinavir from the other two protease inhibitors is probably responsible for its total interaction energy which, as reported in Fig. 4 (Panel C), is quantitatively greater than that computed for Lopinavir and Ritonavir (Figs. 2, 3—Panel C). Intriguingly, this in-silico hypothesis has recently found two independent experimental validations, which have highlighted a mild inhibitory activity of Nelfinavir against the SARS-CoV-2 M^pro^ (estimated between 250 and 600 μM)^25,26^.

## Methods

### Software overview

MOE suite (Molecular Operating Environment, version 2018.0101) was used to perform most of the general molecular modeling operations, such as proteins and ligands preparation^27^. All these operations have been performed on an 8 CPU (Intel Xeon CPU E5-1620 3.50 GHz) Linux workstation. Molecular dynamics (MD) simulations were performed with an ACEMD3 engine on an Nvidia GPU cluster composed of 20 NVIDIA drivers, whose models go from GTX 1080 to Titan V^28^. For all the simulations, the ff14SB force field was adopted to describe C30 Endopeptidase protein while general Amber force field (GAFF) was adopted to parameterize small organic molecules^29–31^.

### Structures preparation

The three-dimensional coordinates of C30 Endopeptidase protein in complex with a covalent peptidomimetic inhibitor (N3) were retrieved from the RCSB PDB database and prepared for SuMD simulations as herein described^32^. Considering the perfect symmetry that characterizes this homodimeric protein, and therefore its two catalytic binding sites, only one of the two monomers was used in this computational investigation. Once the covalent ligand was removed, residue Cys145 was restored to its reduced form. Protein was then processed by means of MOE protein structure preparation tool: residues missing atoms were built according to AMBER14 force field topology. Missing hydrogen atoms were added to X-ray derived complexes and appropriate ionization states were assigned by the Protonate-3D tool^33^. The coordinates of three antiviral compounds were prepared through MOE builder tool and subsequently moved at least 30 Å away from the catalytic protease binding cleft, a distance bigger than the electrostatic cut-off term used in the simulation (9 Å with Amber force field) to avoid premature interaction at the initial phases of the SuMD simulations.

### Solvated system setup and equilibration

Each system investigated by means of SuMD contains a C30 Endopeptidase target macromolecule and respectively one of the three HIV antiviral compounds taken into consideration in this study, moved far away from the protein binding site as previously described. The systems were explicitly solvated by a cubic water box with cell borders placed at least 15 Å away from any protein/ligand atom, using TIP3P as a water model. To neutralize the total charge of each system, Na^+^/Cl^−^ counterions were added to a final salt concentration of 0.154 M. The systems were energy minimized by 500 steps with the conjugate-gradient method, then 500,000 steps (1 ns) of NVE followed by 500,000 steps (1 ns) of NPT simulations were carried out, both using 2 fs as time step and applying harmonic positional constraints on protease and ligands heavy atoms by a force constant of 1 kcal mol^−1^ Å^−2^, gradually reduced with a scaling factor of 0.1. During this step, the temperature was maintained at 310 K by a Langevin thermostat with low dumping of 1 ps^−1^ and the pressure at 1 atm by a Monte Carlo barostat^34^. The M-SHAKE algorithm was applied to constrain the bond lengths involving hydrogen atoms. The particle-mesh Ewald (PME) method was exploited to calculate electrostatic interactions with a cubic spline interpolation and 1 Å grid spacing, and a 9.0 Å cutoff was applied for Lennard–Jones interactions^35^.

### Supervised molecular dynamics (SuMD) simulations

SuMD code, in this implementation, is written in Python and exploits the ProDy python package to perform the geometrical ligand-target supervision process^36^. SuMD protocol reduces the timescale, and consequently the computational effort, required to sample a binding event in the range of nanoseconds, instead of hundreds of nanoseconds or microseconds usually necessary with unbiased MD. Sampling is improved by applying a tabu-like algorithm that monitors the distance between the ligand center of mass with respect to the protein binding site, during short unbiased MD simulations of 600 ps. Once a SuMD step has been collected, the distance points calculated at regular time intervals are fitted into a linear function. Only productive MD steps are maintained, those in which the computed slope is negative, indicating a ligand approach toward the protease catalytic binding site. Otherwise, the simulation is restarted by randomly assigning the atomic velocities. Supervision algorithm controlled the sampling of short simulations until the distance between the ligand and the protein binding site dropped below 5 Å, then was disabled, and a classical MD simulation was performed. For each case study up to a maximum of ten SuMD binding simulations were collected, of which only the best was thoroughly analyzed and discussed in the manuscript.

### SuMD trajectories analysis

All the SuMD trajectories collected were analyzed by an in-house tool written in tcl and python languages, as described in the original publication^19^. Briefly, the dimension of each trajectory was reduced saving MD frames at a 20 ps interval, each trajectory was then superposed and aligned on the protease C_α_ atoms of the first frames and wrapped into an image of the system simulated under periodic boundary condition. The molecular recognition was monitored by calculating for each simulation step the distance between the catalytic binding site and the center of mass of the ligand taken into consideration (Figs. 2, 3, 4—Panel B). A ligand–protein interaction energy estimation during the recognition process was calculated using an NAMD engine, plotting the ligand-receptor interaction energy values over time^37^. These values were also arranged according to the distances between ligand and protease binding site mass centers in the Interaction Energy Landscape plots (Figs. 2, 3, 4—Panel C). Here, the distances between mass centers are reported on the x-axis, while the ligand-receptor interaction energy values on the y-axis, and are rendered by a colorimetric scale going from blue to red for negative to positive energetic values. These graphs allow evaluating the variation of the interaction energy profile at different ligand–protein distances, helping to individuate meta-stable binding states during the binding process. Furthermore, for each target investigated in this work, the residues within a distance of 4 Å from the respective ligand atoms were dynamically selected, to qualitatively and quantitatively evaluated the number of contacts during the entire binding process. The most contacted residues were thus selected, to compute a per-residues electrostatic and vdW interaction energy contribution with the protease target. NAMD was used for post-processing computation of electrostatic interactions, using AMBER ff14SB force field. The total per-residue interaction energy, defined as the sum of vdW and electrostatic per-residue contribution to binding were reported on the Dynamic Total Interaction Energy plots (Figs. 2, 3, 4—Panel D). The cumulative electrostatic interactions were computed for the same target residues by summing the energy values frame by frame along the trajectory, and the resulting graphs were reported at the lower-right of movies provided on supplementary material (Video V1–V3). Representations of the molecular structures were prepared with VMD software^38^.

### SuMD videos

Each video, as accurately described on the work of Salmaso et al., is composed of four synchronized and animated panels that depict the molecular trajectory obtained by the SuMD simulation considering different aspects of the simulation^19^. The time evolution is reported on an ns scale. In the first panel (upper-left), the molecular representation of the SARS-CoV-2 main protease is shown. The protein backbone is represented by the ribbon style (pink color) and the residues within 4 Å of each ligand investigated are shown in green, orange and cyan colors respectively for Lopinavir, Ritonavir, and Nelfinavir. In the second panel (upper-right), the dynamic distance of each ligand center of mass (CM) from the respective protein catalytic binding site during the trajectory is reported. In the third panel (lower-left), the ligand–protein interaction energy profile is reported. The animated red circle highlights the value of the corresponding frame. The trend is depicted by a continuous black line obtained by smoothing the raw data (grey circles) using a Bezier curve procedure. In the fourth panel (lower-right) cumulative electrostatic interactions are reported for the 15 protein residues most contacted by each ligand during the whole simulation.

## Supplementary information

Supplementary information.

Supplementary video V1.

Supplementary video V2.

Supplementary video V3.

## References

[1] Guarner J (2020). Three emerging coronaviruses in two decadesthe story of SARS, MERS, and now COVID-19. Am. J. Clin. Pathol..

[2] Who. *Coronavirus disease (COVID-19) Global epidemiological situation*.

[3] Zhang L, Liu Y (2020). Potential interventions for novel coronavirus in china: a systemic review. J. Med. Virol..

[4] Heymann DL, Shindo N, WHO Scientific and Technical Advisory Group for Infectious Hazards (2020). COVID-19: what is next for public health?. Lancet (London, England).

[5] Mani D, Wadhwani A, Krishnamurthy PT (2019). Drug repurposing in antiviral research: a current scenario. J. Young Pharm..

[6] Gralinski LE, Menachery VD (2020). Return of the coronavirus: 2019-nCoV. Viruses.

[7] Keener AB (2020). Four ways researchers are responding to the COVID-19 outbreak. Nat. Med..

[8] Letko M, Munster V (2020). Functional assessment of cell entry and receptor usage for lineage B β-coronaviruses, including 2019-nCoV. bioRxiv.

[9] Wrapp D (2020). Cryo-EM structure of the 2019-nCoV spike in the prefusion conformation. Science.

[10] Anand, K., Yang, H., Bartlam, M., Rao, Z. & Hilgenfeld, R. Coronavirus main proteinase: target for antiviral drug therapy. in *Coronaviruses with Special Emphasis on First Insights Concerning SARS* 173–199 (Birkhäuser-Verlag, 2005). 10.1007/3-7643-7339-3_9.

[11] Li Y (2020). Therapeutic drugs targeting 2019-nCoV main protease by high-throughput screening. bioRxiv.

[12] Xu Z (2020). Nelfinavir was predicted to be a potential inhibitor of 2019-nCov main protease by an integrative approach combining homology modelling, molecular docking and binding free energy calculation. bioRxiv.

[13] Liu X, Wang X-J (2020). Potential inhibitors for 2019-nCoV coronavirus M protease from clinically approved medicines. bioRxiv.

[14] Contini, A. *Virtual Screening of an FDA Approved Drugs Database on Two COVID-19 Coronavirus Proteins*. 10.26434/CHEMRXIV.11847381.V1 (2020).

[15] Chu CM (2004). Role of lopinavir/ritonavir in the treatment of SARS: Initial virological and clinical findings. Thorax.

[16] Sheahan TP (2020). Comparative therapeutic efficacy of remdesivir and combination lopinavir, ritonavir, and interferon beta against MERS-CoV. Nat. Commun..

[17] Sabbadin D, Moro S (2014). Supervised molecular dynamics (SuMD) as a helpful tool to depict GPCR–ligand recognition pathway in a nanosecond time scale. J. Chem. Inf. Model..

[18] Cuzzolin A (2016). Deciphering the complexity of ligand-protein recognition pathways using supervised molecular dynamics (SuMD) simulations. J. Chem. Inf. Model..

[19] Salmaso V, Sturlese M, Cuzzolin A, Moro S (2017). Exploring protein-peptide recognition pathways using a supervised molecular dynamics approach. Structure.

[20] Yamamoto N (2004). HIV protease inhibitor nelfinavir inhibits replication of SARS-associated coronavirus. Biochem. Biophys. Res. Commun..

[21] Cvetkovic RS, Goa KL (2003). Lopinavir/ritonavir: a review of its use in the management of HIV infection. Drugs.

[22] Cheng SC, Chang GG, Chou CY (2010). Mutation of glu-166 blocks the substrate-induced dimerization of SARS coronavirus main protease. Biophys. J..

[23] Ho BL (2015). Critical assessment of the important residues involved in the dimerization and catalysis of MERS Coronavirus Main Protease. PLoS ONE.

[24] Douangamath A (2020). Crystallographic and electrophilic fragment screening of the SARS-CoV-2 main protease. bioRxiv.

[25] Ghahremanpour MM (2020). Identification of 14 known drugs as inhibitors of the main protease of SARS-CoV-2. bioRxiv.

[26] Vatansever EC (2020). Targeting the SARS-CoV-2 main protease to repurpose drugs for COVID-19. bioRxiv Prepr. Serv. Biol..

[27] Chemical Computing Group (CCG) Inc. *Molecular Operating Environment (MOE)* (2018).

[28] Harvey MJ, Giupponi G, De Fabritiis G (2009). ACEMDL: accelerating biomolecular dynamics in the microsecond time scale. J. Chem. Theory Comput..

[29] Tan D, Piana S, Dirks RM, Shaw DE (2018). RNA force field with accuracy comparable to state-of-the-art protein force fields. Proc. Natl. Acad. Sci. U. S. A..

[30] Wang J, Wang W, Kollman PA, Case DA (2006). Automatic atom type and bond type perception in molecular mechanical calculations. J. Mol. Graph. Model..

[31] Sprenger KG, Jaeger VW, Pfaendtner J (2015). The general AMBER force field (GAFF) can accurately predict thermodynamic and transport properties of many ionic liquids. J Phys Chem B.

[32] Berman HM (2000). The protein data bank. Nucleic Acids Res..

[33] Labute P (2009). Protonate3D: assignment of ionization states and hydrogen coordinates to macromolecular structures. Proteins.

[34] Loncharich RJ, Brooks BR, Pastor RW (1992). Langevin dynamics of peptides: the frictional dependence of isomerization rates ofN-acetylalanyl-N?-methylamide. Biopolymers.

[35] Essmann U (1995). A smooth particle mesh Ewald method. J. Chem. Phys..

[36] Bakan A, Meireles LM, Bahar I (2011). ProDy: protein dynamics inferred from theory and experiments. Bioinformatics.

[37] Phillips JC (2005). Scalable molecular dynamics with NAMD. J. Comput. Chem..

[38] Humphrey W, Dalke A, Schulten K (1996). VMD—visual molecular dynamics. J. Mol. Graph..

