# Supplementary material for: Targeting the coronavirus SARS-CoV-2: computational insights into the mechanism of action of the protease inhibitors lopinavir, ritonavir and nelfinavir
Source: Sci Rep. 2020 Dec 1;10:20927. doi: 10.1038/s41598-020-77700-z (PMC7708625; doi:10.1038/s41598-020-77700-z)
Supplement: Supplementary file 1 — Supplementary information. [file 41598_2020_77700_MOESM1_ESM.docx]

**Targeting the Coronavirus SARS-CoV-2: computational insights into the mechanism of action of the protease inhibitors Lopinavir, Ritonavir and Nelfinavir.**

Giovanni Bolcato, Maicol Bissaro, Matteo Pavan, Mattia Sturlese and Stefano Moro*

*Molecular Modeling Section (MMS), Department of Pharmaceutical and Pharmacological Sciences University of Padova, Via Marzolo5, 35131Padova (Italy)*

**SUPPLEMENTARY FIGURES**

**Supplementary Figure 1**

**
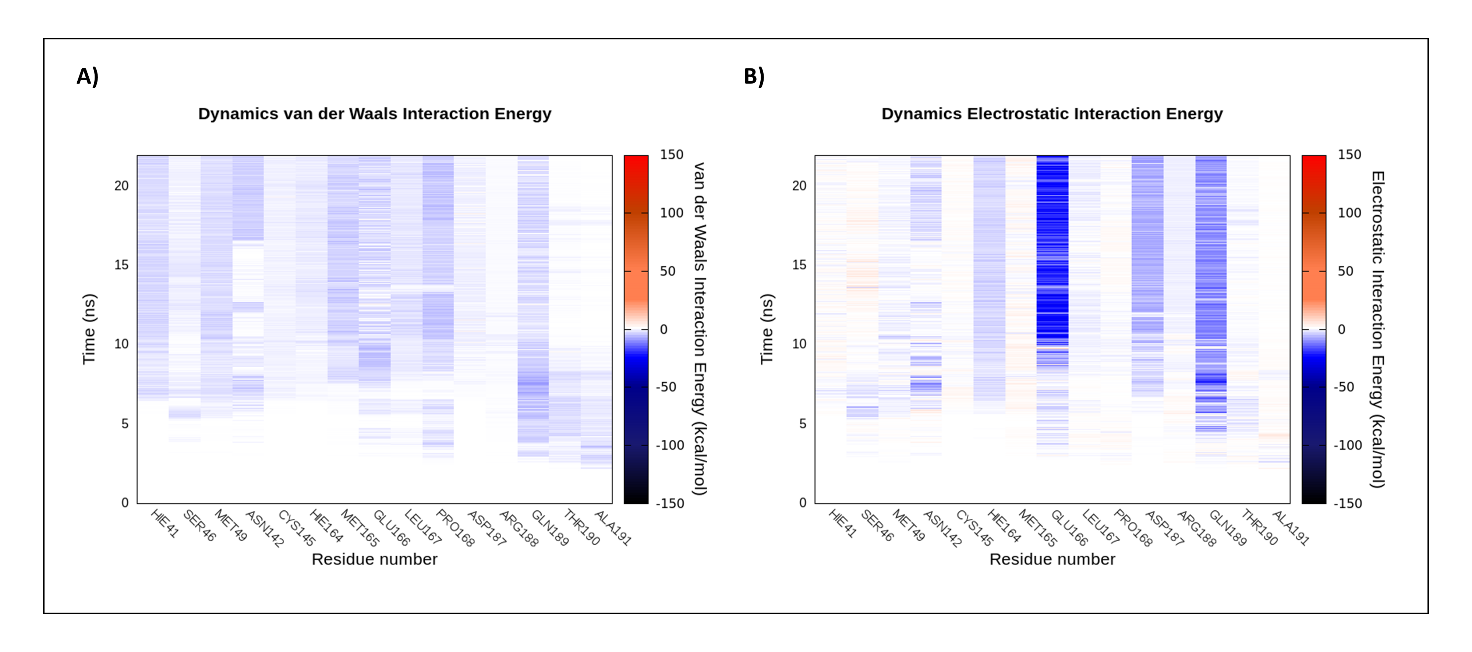
**

**Figure S1** Time-dependent per-residue analysis of the van der Waals (Panel A) and electrostatic (Panel B) contribution to the binding interaction energy for the Lopinavir SuMD simulation.

**Supplementary Figure 2**

**
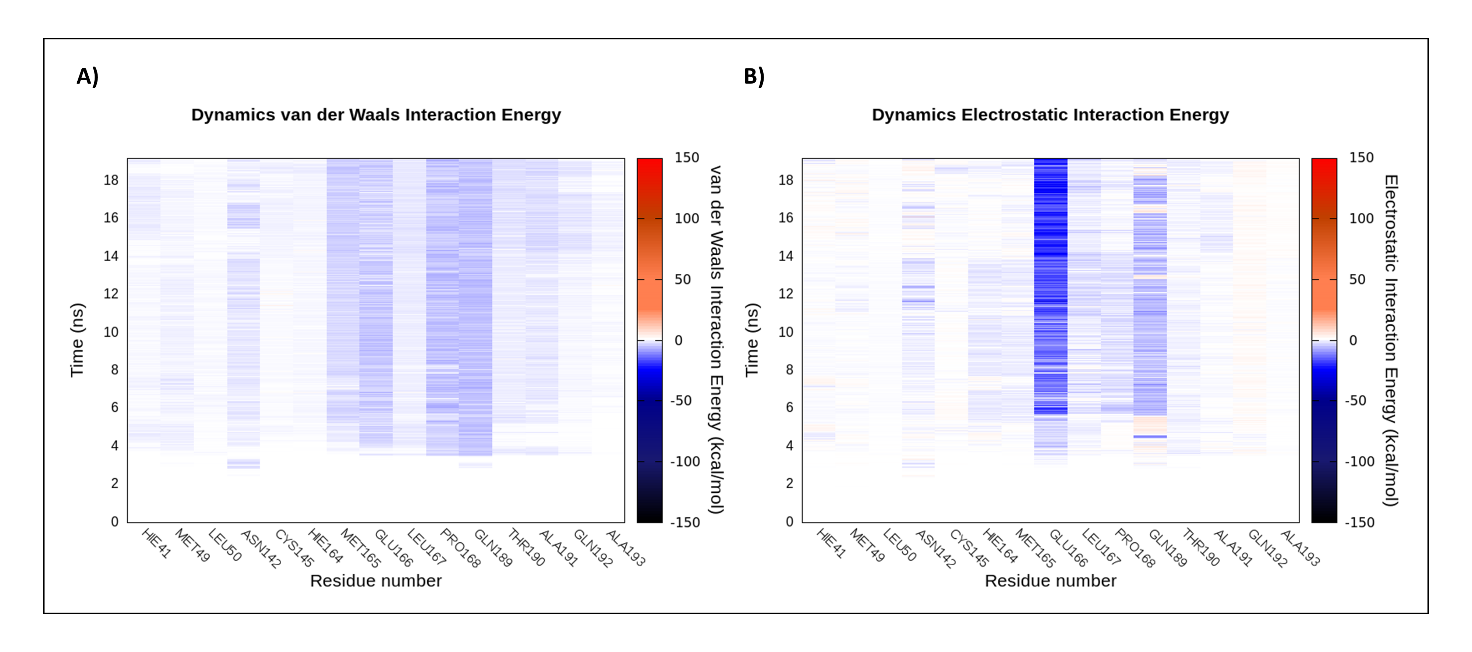
*Figure S2*** *Time-dependent per-residue analysis of the van der Waals (Panel A) and electrostatic (Panel B) contribution to the binding interaction energy for the Ritonavir SuMD simulation.*

**
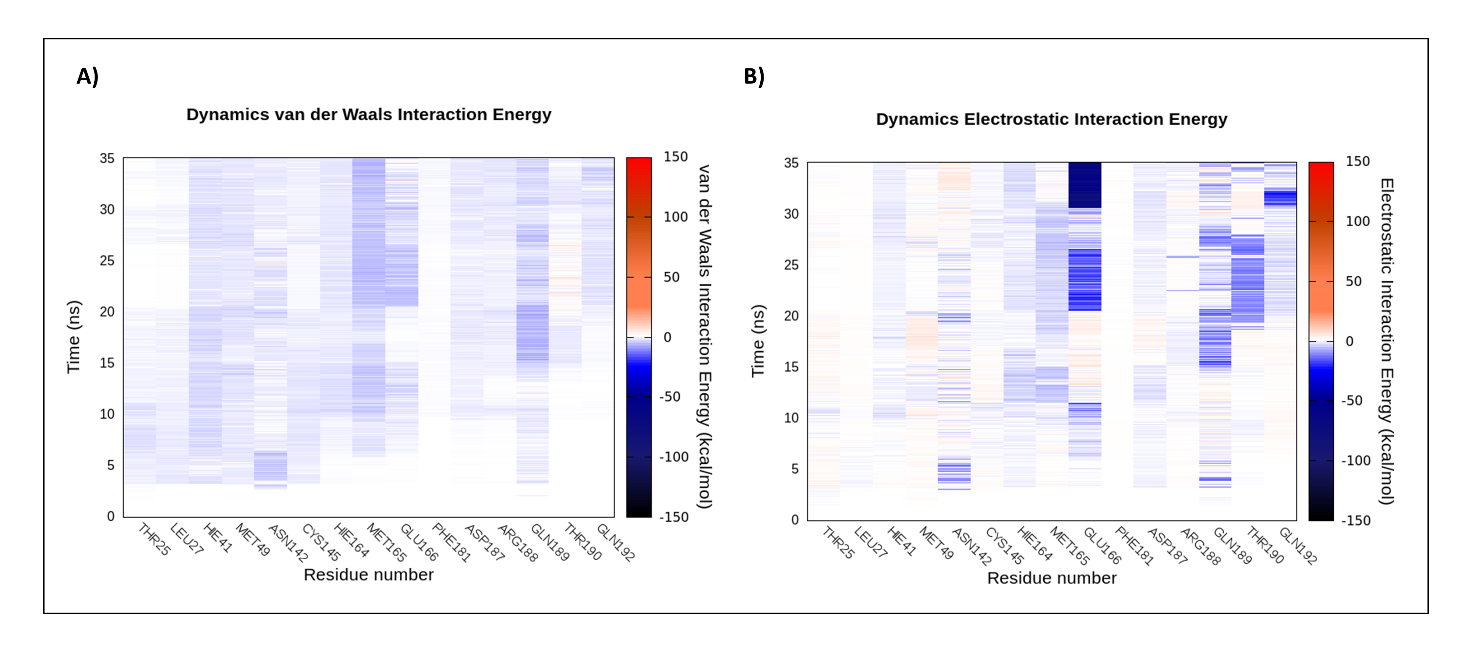
**

**Figure S3** Time-dependent per-residue analysis of the van der Waals (Panel A) and electrostatic (Panel B) contribution to the binding interaction energy for the Nelfinavir SuMD simulation.
